# A European survey on the conservative surgical management of endometriotic cysts on behalf of the European Society for Gynaecological Endoscopy (ESGE) Special Interest Group (SIG) on Endometriosis

**Published:** 2020-08-05

**Authors:** G Pados, A Daniilidis, J Keckstein, P Papandreou, S Gordts

**Affiliations:** 1st Dept. OB-GYN, “Papageorgiou” General Hospital, Aristotle University of Thessaloniki and Centre for Endoscopic Surgery “Diavalkaniko” Hospital, Thessaloniki, Greece;; 2nd Dept. OB-GYN, ‘Hippokratio’ General Hospital, Aristotle University of Thessaloniki, Greece;; Landeskrankenanstalten-Betriebsgesellschaft (KABEG) and Landeskrankenhaus Villach, Abteilung fur Gynakologie und Geburtshilfe, Villach, Austria;; Life Expert Centre, Leuven, Belgium.

**Keywords:** Endometrioma, laparoscopic surgery, cystectomy, stripping, ablation, excision

## Abstract

The mainstay of endometrioma management, when treatment is required, is surgical. Although laparoscopy is considered to be the gold standard for endometriosis surgery, there is no clarity on the preferred laparoscopic technique, which may depend on whether the primary goalis treatment of infertility or pelvic pain, prevention of recurrence or preservation of ovarian reserve.

The aim of this survey to assess the surgical practice of the members of the European Society for Gynaecological Endoscopy (ESGE) on the conservative management of endometiotic cysts in women of reproductive age.

The current survey showed that practice for the conservative management of endometriotic cysts was that laparoscopy accounted for 84.9% of the cases, expectant management for 12.1%, and laparotomy for 3%. The preferred surgical approach was cystectomy in 69% of the cases, while the parameters that determined the preferred surgical method were the diameter of the cyst (62%) and the bilaterality or non-location (53%). The type of energy used was in most cases bipolar (83%), 71.4% of surgeons did not reconstitute the ovary and 41% of responses included the administration of adhesion barrier agents. The primary surgical end-point was ovarian reserve (50%), which was tested preoperatively in 51.8%, mainly with an anti-mullerian hormone. In case of an incidentally deep-infiltrating endometriosis, 55.4% of the responses included concomitant treatment thereof, while 71% of the participants considered that a “pelvic surgeon”, who could more effectively treat co- existing pelvic and intestinal disease, should be the ideal one to effectively manage endometriosis.

The majority of participants (74%) in this survey consider that there is insufficient scientific evidence regarding the conservative management of endometriotic cysts. The treatment of ovarian endometrioma should be individualised, taking into consideration not only the relief of symptoms, pregnancy rates or recurrence rates, but also ovarian function and reserve after surgery.

## Introduction

Endometriosis is a chronic condition that affects 5-10% of women of reproductive age (Nickkho- Amiry et al., 2018). It is characterised by the presence of endometrial tissue outside the uterus, which induces a chronic inflammatory reaction and the formation of scar tissue and adhesions, resulting in deformation of the female pelvic anatomy (Dunselman et al., 2014; Nickkho-Amiry et al., 2018). Endometriosis may be associated with a long list of gynaecological symptoms (dysmenorrhoea, non-cyclical pelvic pain, deep dyspareunia, infertility, and fatigue in the presence of any of the above) and non-gynaecological cyclical symptoms (dyschezia, dysuria, haematuria, rectal bleeding, and shoulder pain) (Dunselman et al., 2014). Endometrioma is a benign cyst of the ovary containing ectopic endometrial tissue, and most endometriomas are pseudocysts with their pathogenesis being different from other benign ovarian cysts (Nickkho-Amiry et al., 2018). They are probably the most commonly diagnosed form of the disease due to the relative ease and accuracy of the ultrasonographic diagnosis (Saridogan et al., 2017), and often co-exist with deep infiltrating endometriosis (Nickkho-Amiry et al., 2018). The mainstay of endometrioma management, when treatment is required, is surgical (Saridogan et al., 2017). For women of reproductive age who wish to conceive, surgical treatment should be conservative to preserve the normal ovarian tissue and blood supply (Saridogan et al., 2017; Nickkho-Amiry et al., 2018) and laparoscopy has been proven as the ideal tool for diagnosis and treatment (Yeung et al., 2009). Conservative surgical treatment methods include cystectomy (excision of the cyst capsule), drainage of the cyst content and laser or plasma energy ablation, or electrocoagulation, combined technique (both excision of the cyst wall and ablation) and the “three-step” approach, with 3-month administration of GnRH analogue between the two laparoscopic interventions, for the treatment of large endometriomas (Hamdan et al., 2015a). For infertile women with endometriosis undergoing an assisted reproductive cycle, the American Society of Reproductive Medicine (ASRM) stage of the disease seems to be a considerable factor, affecting reproductive outcome compared to women without the disease (Harb et al., 2013; Hamdan et al., 2015b). Early endometriosis (ASRM stage I-II) does not seem to affect implantation, clinical pregnancy and live birth rates in women undergoing in vitro fertilization, whereas more severe disease (ASRM stage III-IV) has an effect on fertility (Harb et al., 2013). In addition, for women with endometrioma undergoing in vitro fertilisation, the presence of the disease in the ovaries does not appear to affect clinical pregnancy and live birth rates; however, it is associated with fewer numbers of retrieved oocytes and higher cycle cancellation rates compared to women without the disease (Tsoubou et al, 2009; Hamdan et al., 2015b). On the other hand, surgical treatment of endometrioma increases the risk of reduced ovarian function or loss of the ovary (Dunselman et al., 2014; Schleedoorn et al., 2016; Nickkho-Amiry et al., 2018; Younis et al., 2019).

The aim of this survey was to determine the surgical practice of the members of the European Society for Gynaecological Endoscopy (ESGE) on the conservative management of endometiotic cysts in women of reproductive age.

## Materials and methods

The project was approved by the Executive and Advisory Boards of ESGE and invitation letters were sent to all members of ESGE to participate in the 22-item online questionnaire accessible via Survey Monkey. From June 1 to July 15, 2018, website visitors were able to fill in the questionnaire. Financial incentives were not presented to the survey participants.

## Results

Sixty-six gynaecologists, mostly from Europe, participated in the survey Fifty-five (83.3%) of the participants were specialists and 11 (16.7%) were trainees. Among the participants, the clinical practice period ranged from 1 to 35 years, and the number of laparoscopic surgeries ranged from 0 to 10,000. Forty-eight (72.7%) of the gynaecologists had more than 10 years of clinical practice experience and 43 (65.2%) of them performed more than 100 laparoscopic interventions per year. The majority of them (60.6%) were based at university hospitals and 39.4% worked in a national or private health system hospital. Their preferred practice for the conservative management of endometriotic cysts was laparoscopy for 84.9% of the cases, expectant management for 12.1% and laparotomy for 3.0%. 75.8% of the participants did not recommend medical treatment with combined hormonal contraceptives, dienogest, progestogens or GnRH analogues prior to the surgical approach. The preferred surgical approach included cystectomy for 68.0% , combined technique (excision combined with ablative surgery close to the hilus) for 12.5%, fenestration and electrocoagulation of the cyst capsule for 10.7%, “three-stage” management for 5.4% and other techniquesfor 3.6%. The type of energy used for the conservative laparoscopic treatment of a cyst included bipolar electricity accounting for 83.9%, monopolar for 8.9%, plasma energy for 5.4% and CO 2 laser for 1.8% of the cases. Factors that influenced the preferred surgical method were the diameter of the cyst (62.5%), bilaterality (53.5%), low ovarian reserve (51.7%), previous ovarian surgery for endometriosis (44.6%) and age ≥ 37 years (26.8%). The primary surgical end-point for conservative management of endometriotic cysts was the ovarian reserve (50%) and disease recurrence (50%). Ovarian reserve was evaluated before surgery by 51.8% of the participants, and as the most reliable test for predicting ovarian reserve was serum AMH(91.7%) and antral follicle count (AFC, 53.57%). The majority of participants (63.4%) correlated the presence of endometrioma with a significant increase in the incidence of deep infiltrating endometriosis (DIE), while 55.4% responded that they treated incidental DIE during conservative surgery for endometrioma. 62.5% of the participants prescribed oral contraceptive pills and 21.43% used GnRH-analogues postoperatively, in order to reduce the risk of long-term recurrence of the disease. According to 71.4% of the participants, a pelvic surgeon who can effectively treat extensive pelvic and intestinal disease should be the ideal one to manage laparoscopic endometrial cysts. Finally, 73.2% considered the scientific evidence on the conservative management of endometriotic cyst was insufficient.

## Discussion

Laparoscopy is an ideal tool for the diagnosis and treatment of endometriosis due to the benefits of magnification and illumination (Yeung et al., 2009). It is beneficial in reducing endometriosis-related pain and improving infertility, and is usually associated with less postoperative pain, shorter hospital stay, and quicker recovery, as well as a better cosmetic outcome (Yeung et al., 2009; Dunselman et al., 2014). According to the participants’ responses, although the number of received questionnaires is a major limitation of the study, the current practice for the conservative management of endometriotic cysts was laparoscopy in the majority of the cases. Most participants did not recommend preoperative medical treatment with combined hormonal contraceptives, dienogest, progestogenes or GnRH analogues to prevent surgical complications, facilitate surgery or reduce the risk of recurrence and this is consistent with published national guidelines from Europe (Collinet et al., 2018). The preferred surgical approach was cystectomy in most cases, followed by the “combined” technique.

Cystectomy prevails as a method for conservative surgical treatment of endometrioma, as it is associated with postoperative reduction in endometriosis-related pain, a lower recurrence rate, and an increased spontaneous pregnancy rate compared to other surgical techniques, and this is in accordance with the literature (Yeung et al., 2009; Dunselman et al., 2014). Compared with electrocoagulation, laser or plasma energy ablation seems to prevail in haemostasis, prevent excessive bleeding, and prevent damaging the ovarian hilum tissue (Saridogan et al., 2017). It is suggested that electrocoagulation, during conservative surgical treatment of endometrioma, should be carefully applied to avoid deep tissue injury and limit damage to healthy ovarian tissue, primordial follicles and ovarian vessels. The use of bipolar diathermy seems to be a safer method for coagulation of endometriosis compared to monopolar diathermy (Saridogan et al., 2017). The combined surgical technique is recommended for the treatment of large endometriomas, in order to prevent excessive bleeding or tissue damage from the ovarian hilum (Saridogan et al., 2017). The “three-step” approach is also put forward for the treatment of larger endometriomas and is associated with a limited decrease in ovarian reserve and lower recurrence rates (Pados et al., 2010; Tsolakidis et al., 2010; Saridogan et al., 2017). For women of reproductive age who wish to conceive, surgical treatment should be conservative to achieve a diagnostic histological evaluation and to eliminate endometriotic tissue, thereby preserving the normal ovarian tissue and blood supply (Saridogan et al., 2017; Nickkho-Amiry et al., 2018). Surgical treatment of endometrioma increases the risk of reduced ovarian function or loss of the ovary (Dunselman et al., 2014; Schleedoorn et al., 2016; Saridogan et al., 2017; Younis et al., 2019) and the results of the present survey are in line with the published literature. Postoperative reduction in ovarian reserve is associated with low-serum anti- Mullerian hormone (AMH) levels, reduced antral follicle count (AFC) on ultrasound, fewer numbers of retrieved oocytes, and greater risk of failure of controlled ovarian hyperstimulation during an assisted reproduction cycle (Hamdan et al., 2015b; Nickkho-Amiry et al., 2018; Younis et al., 2019). Surgical treatment for bilateral endometriotic cysts is associated with a greater reduction in ovarian reserve compared to unilateral endometrioma excision (Younis et al., 2019). Serum AMH levels can be significantly decreased by up to 57% after resection of bilateral endometriotic cysts (Younis et al., 2019).

The majority of participants in this survey did not reconstitute the ovary by suturing after conservative laparoscopic management of endometrioma and 41.1% administered adhesion barrier agents to reduce adhesion formation postoperatively. Suturing may be necessary to reconstruct the ovary and achieve haemostasis after removal of large endometriomas. Monofilament sutures should ideally be placed inside the ovary to prevent adhesion formation (Saridogan et al., 2017). For the prevention of postoperative adhesions, although it is not a substitute for good surgical techniques, the use of adhesion barriers is recommended to be safe and effective (Dunselman et al., 2014). It is highly recommended that ovarian reserve is preoperatively assessed by serum AMH or AFC and the findings of this survey are consistent with this (Saridogan et al., 2017). Fertility preservation for these women is of utmost importance when the ovarian reserve has already been compromised, since surgical treatment of endometriomas is associated with an even greater reduction in AMH levels and AFC (Hamdan et al., 2015b; Nickkho-Amiry et al., 2018; Younis et al., 2019). Endometriotic cysts often co-exist with DIE (Nickkho-Amiry et al., 2018).The majority of participants (73.2%) evaluated the presence of kissing ovaries (endometriotic cysts in both ovaries joined together and stabilised behind the uterus) as a marker of the most severe case of the disease and this is in accordance with the literature (Keckstein et al., 2019). Surgical treatment for bilateral endometriotic cysts is associated with a greater reduction in ovarian reserve compared to unilateral endometrioma excision (Younis et al., 2019). Oral contraceptives, levonorgestrel-releasing intrauterine device or GnRH analogues seem to be beneficial in preventing disease recurrence and improving quality of life, and is therefore recommended for use when pregnancy is not desired after surgery (Seo et al., 2017; Collinet et al., 2018) and this view was shared by the majority of the participants, of whom 62.5% used GnRH-a postoperatively. Since ovarian endometriosis is often associated with DIE, a multidisciplinary surgical team, involving a bowel surgeon, an urologist, a thoracic surgeon alongside the gynaecologist, should be organised according to the planned procedure (Keckstein et al., 2019). Alternatively, an adequately trained gynaecologist or a “pelvic surgeon” who can effectively treat extensive pelvic and intestinal disease as well as endometriotic cysts, may be the ideal surgeon to manage endometriosis, although this issue is still debatable.

## Conclusion

The findings of this survey support that laparoscopic cystectomy is the first choice for conservative treatment of endometriotic cysts and that treatment of ovarian endometrioma should take into consideration not only the relief of symptoms, pregnancy rates or recurrence rates, but also the ovarian function and reserve after surgery. The majority of gynaecologists, who participated in this survey, consider a pelvic surgeon as the ideal person to manage endometriosis and affirm that the scientific evidence regarding the conservative management of endometriotic cysts is still insufficient. There is a need for well-designed research with validated and detailed data to provide clear answers.
